# Vulnerable Dignity, Dignified Vulnerability: Intertwining of Ethical Principles in End-of-Life Care

**DOI:** 10.3390/ijerph18020482

**Published:** 2021-01-09

**Authors:** José María Muñoz Terrón

**Affiliations:** Departament of Geography, History and Humanities, University of Almería, La Cañada de San Urbano, E-04120 Almería, Spain; jmterron@ual.es; Tel.: +34-950-01-52-24

**Keywords:** bioethics, ethical principles, autonomy, dignity, vulnerability, corporeality, responsiveness, end-of-life care

## Abstract

The aim of this article is to analyze how dignity and vulnerability, as declared principles of bioethics, both can be seen in a new light when they are thought of together, in their intertwining, in order to outline a proposal for an analytical framework for end-of-life care. It is thus shown, on the one hand, that the demand for respect for the equal dignity of every person, linked by the different anthropological and ethical theories to their autonomy as a rational agent, also refers to their fragile, vulnerable, and interdependent character, as an embodied subjectivity, sustained by a complex web of care. On the other hand, the vulnerability of these selves as others, constituted by the radical appeal of everything that affects them socially, emotionally, sensitively, and by their need for recognition and attention, would be pathological if it did not include the impulse towards autonomy, which, although precarious and connotative, requires dignified and equitable treatment. This intertwining of both principles points to a phenomenological conception of the person as a corporeal social existence, from which a number of studies on the attention to dignity and vulnerability at the end of life are analyzed.

## 1. Introduction

The aim of this study is to present dignity and vulnerability as two fundamental ethical principles of care at the end of life, which acquire full meaning when they are thought of together, showing their intertwining. Firstly, the presence, latent or express, of both principles is examined in various bioethical documents of international importance, from the influential Belmont Report in the United States to the UNESCO Declaration on Bioethics and Human Rights. Next, on one hand, the study proposes that the recognition of vulnerability is already implicit in the demand for respect for dignity, linked by the philosophical tradition to the rational autonomy of the person (from stoicism, passing through the humanism of Pico della Mirandola, to the enlightened ethics of Immanuel Kant) since it does not deal with a sovereign or autarkic subject but rather fragile, vulnerable, and interdependent beings who are in need of care. On the other hand, it also shows how vulnerability, as a dimension of the human condition and of a self constituted by the appeal of otherness (E. Levinas), can only be understood as an ethical principle, as due to their precariousness fragile existences feel driven to realize themselves as co-protagonist agents of their lives, aware of the dignity of their own value (P. Ricoeur). From this double perspective, of a vulnerable dignity and dignified vulnerability, the study points to a responsive phenomenological conception of the person as an intercorporeally situated social and corporeal existence (M. Merleau-Ponty, B. Waldenfels), whose importance as a theoretical framework to investigate the care at the end of life is shown through the analysis, by way of example, of certain studies on the perception of dignity and vulnerability by health personnel, patients, or family members in health care contexts.

## 2. Dignity and Vulnerability in the Declarations of the Principles of Bioethics

In the late 1970s, the findings of The National Commission for the Protection of Human Subjects of Biomedical and Behavioral Research were released in the form of a statement of Ethical Principles and Guidelines for the Protection of Human Subjects of Research, better known as the Belmont Report [1,2]. In this document, both dignity and vulnerability are present, more the former than the latter, although neither of the two is expressly mentioned.

Dignity appears associated with the principle that the declaration initially states as “respect for persons”, later called the principle of autonomy, and which, according to the Belmont Report, “incorporates at least two ethical convictions: first, that individuals should be treated as autonomous agents, and second, that persons with diminished autonomy are entitled to protection”. In other words, this principle of respect for people as autonomous beings leads to differentiate “two separate moral requirements: the requirement to acknowledge autonomy and the requirement to protect those with diminished autonomy”. Thus, of the two ethical convictions brought together in this principle, the first connects with the dignity of people through the recognition of their autonomy, and the second with the attention to or care for vulnerability due to the requirement for protection of those who they may find their capacity for self-determination diminished. So, as this declaration understands that “[a]n autonomous person is an individual capable of deliberation about personal goals and of acting under the direction of such deliberation”, it follows that “[t]o respect autonomy is to give weight to autonomous persons’ considered opinions and choices while refraining from obstructing their actions unless they are clearly detrimental to others”. Thus, put in negative terms, “[t]o show lack of respect for an autonomous agent is to repudiate that person’s considered judgments, to deny an individual the freedom to act on those considered judgments, or to withhold information necessary to make a considered judgment, when there are no compelling reasons to do so.” Consequently, vulnerability significantly also appears implicit at the very heart of this principle of respect for the dignity of persons, in the form of limitations to the exercise of autonomy, here called self-determination, found by people who are still in the process of maturing, or when “some individuals lose this capacity wholly or in part because of illness, mental disability, or circumstances that severely restrict liberty” [1,2]. One can see in this demand of the Belmont Report for extra protection for dignity in certain situations, a first indication of the future inclusion of vulnerability among the principles of bioethics. In some ways, a connection with vulnerability can also be seen in the other principles declared by the Belmont Report: non-maleficence, beneficence and justice, [3,4] (p. 19).

Vulnerability was specifically mentioned in the so-called Barcelona Declaration (1998), bringing together the concerns of the Belmont Report: “Vulnerability expresses two basic ideas. (a) It expresses the finitude and fragility of life which, in those capable of autonomy, grounds the possibility and necessity for all morality. (b) Vulnerability is the object of a moral principle requiring care for the vulnerable. The vulnerable are those whose autonomy or dignity or integrity is capable of being threatened” [5,6].

More recently, right at the beginning of the 21st century, the BIOMED II Project put forward a proposal for bioethical principles at the European level, the Basic Ethical Principles in European Bioethics and Biolaw [7,8], in which dignity and vulnerability already appear along with autonomy and integrity expressly mentioned as two of the four basic ethical principles.

Likewise, the Universal Declaration on Bioethics and Human Rights, from UNESCO (19 October 2005) also explicitly includes them among the principles that it lists as “Dignity and human rights” (in Article 3) and “Respect for human vulnerability and personal integrity” (in Article 8). Respect for dignity is linked to respect for human rights and fundamental freedoms, indicating the priority of the interests and well-being of people over the exclusive interests of science and society. In terms of vulnerability, reference is made to the application and promotion of scientific knowledge, medical practice and related technologies, and expressly refers to the protection of particularly vulnerable individuals and groups, as well as respect for the personal integrity of the former.

## 3. Dignified and Autonomous in Vulnerability

Dignity and autonomy are grouped together and are closely related in the Barcelona Declaration. Five aspects of autonomy are proposed: (1) capacity of creation of ideas and goals for life; (2) capacity of moral insight, “self-legislation”, and privacy; (3) capacity of decision and action with lack of outer constraint; (4) capacity of political involvement and personal responsibility; and (5) capacity of informed consent. Autonomy is linked to vulnerability when it is pointed out that “autonomy remains merely an ideal because of the structural limitations given to it by human weakness and dependence on biological, material and social conditions, lack of information for reasoning etc.” [5,7] (p. 25).

Dignity, in turn, appears in the Declaration of Barcelona, with its own characteristics, which are not reduced to autonomy. “Although originally a virtue of outstanding persons and a virtue of self-control in healthy life qualities, which can be lost, for instance by lack of responsibility or in extreme illness it has been universalized as a quality of the person as such. It now refers to both the intrinsic value of the individual and the inter-subjective value of every human being in its encounter with the other. Dignity concerns both oneself and the other: I must behave with dignity, and I must consider the dignity of the other; I must not give up civilised and responsible behaviour, and the other should not be commercialised and/or enslaved” [5,7].

At a certain point, the discussion about dignity began to see it as a bombastic and empty term that would not in any concrete way help to make decisions from the ethical and legal point of view in biomedical and health practice [9]. However, this is really a discussion which is the result of a lack of differentiation between two uses of the concept of dignity. On the one hand, the importance of dignity as a policy principle stands out when it is considered from the distinction between principles and rules; on the other hand, dignity is shown as a moral standard of the quality of health care. It is here where the link with vulnerability appears again as it is precisely in the situation of *patients*, as the word itself indicates, where the achievements and shortcomings of health care in treating them as people with all the consequences thereof are most evident [10,11].

In Western philosophical thought, the principle of dignity has explicit historical precedents since Antiquity, but this is not the case with vulnerability, which has been formulated as such much more recently, not only in bioethics but also, as we shall see, in anthropology and moral philosophy. And yet, one can also see how the fragile and precarious character of dignity also tacitly appears from the first formulations.

“The idea of universal respect for the dignity of humanity in each and every person, regardless of class, gender, race, and nation—an idea that has ever since been at the heart of all distinguished political thought in the Western tradition—is, in origin, a Stoic idea” [12] (p. 12). By establishing a clear distinction between humans and animals, Stoicism considers that it is “(t)he presence of reason in any creature entitles it to respect from others, and also from itself” [12] (p. 325).

Lucius A. Seneca believes that the consideration of the supreme and unique value of virtue, its autarky and self-sufficiency, is united with this dignity of every human being through the primacy of reason since virtue is the only thing needed to live well and happily. “Praise the quality in him which cannot be given or snatched away, that which is the peculiar property of the man. Do you ask what this is? It is soul, and reason brought to perfection in the soul. For man is a reasoning animal. Therefore, man’s highest good is attained, if he has fullfilled the good for which nature designed him at birth. […] To live in accordance with his own nature” [13] (XLI, 7–9).

Martha C. Nussbaum (2008) has questioned this Stoic vision, pointing out two serious problems. On the one hand, the Stoic vision of dignity is based on an extreme disjunction of human rationality with respect to non-human animals, which completely devalues all these elements (emotion, sensitivity, affections), that in human life connect with other living beings. On the other hand, Stoic dignity seems to aspire to an invulnerable autarky, which would lead it to disparage as “external goods” all those vital resources and supports that are not the moral virtue itself [14].

The Spanish philosopher María Zambrano, who spent a long time in exile after the 1936–1939 Spanish Civil War, points out that in Seneca this natural reason is not the imperative reason but rather a helpless reason, whose dignity is charged with resignation, because it is “the reason that is not differentiated from life, coinciding with it, and thus it does not serve to explain it, nor to transcend it, much less to endure it. […] Endure life. Carry on with it with dignity. Dignity is the only opening for the Stoic, the closest thing to personal freedom, but more moving in our eyes because it has no horizon; *desperate dignity*”. Now, a “despair not closed to hope”, a resignation that “is neither a believing nor a not believing”, but rather a “yielding, a yielding before death. […] It is not wanting to alter the order of the world for anything, however strange it may be; to look at oneself without resentment, to have ceased to see and feel oneself as something that *is*. […] It is a kind of weakness before the cosmos; to be defeated by it without resentment” [15] (pp. 83–84).

This desperate stoic dignity can be seen in certain passages of *Ad Lucilium Epistulae morales*, in which Seneca considers just how to take care of himself in old age and how to face the perspective of the proximity of his own death. “Do not hear me with reluctance, as if my statement applied directly to you, but weigh what I have to say. It is this: that I shall not abandon old age, if old age preserves me intact for myself, and intact as regards the better part of myself; but if old age begins to shatter my mind, and to pull its various faculties to pieces, if it leaves me, not life, but only the breath of life, I shall rush out of a house that is crumbling and tottering. I shall not avoid illness by seeking death, as long as the illness is curable and does not impede my soul. I shall not lay violent hands upon myself just because I am in pain; for death under such circumstances is defeat. But if I find out that the pain must always be endured, I shall depart, not because of the pain, but because it will be a hindrance to me as regards all my reasons for living. He who dies just because he is in pain is a weakling, a coward; but he who lives merely to brave out this pain, is a fool” [13] (LVIII, 35–36).

In the Renaissance humanism of Giovanni Pico della Mirandola (1486), human dignity is linked to a capacity for self-determination that, although received by the creature from the hands of their Creator, depends above all on their own free endeavor [16], from which the best [17] or the worst may result. “The nature of all other creatures is defined and restricted within laws which we have laid down; you, by contrast, impeded by no such restrictions, may, by your own free will, to whose custody We have assigned you, trace for yourself the lineaments of your own nature […]. We have made you a creature neither of heaven nor of earth, neither mortal nor immortal, in order that you may, as the free and proud shaper of your own being, fashion yourself in the form you may prefer. It will be in your power to descend to the lower, brutish forms of life; you will be able, through your own decision, to rise again to the superior orders whose life is divine” [18] (pp. 7–8).

But perhaps the most extreme union of dignity and vulnerability is represented by the paradoxical baroque thought of Blaise Pascal (1670), who continues to associate the greatness (dignity) of the little human being, beset by all kinds of dangers in the universe, to his thinking capacity, to his perceiving his own vulnerability: “Man is but a reed, the most feeble thing in nature; but he is a thinking reed. The entire universe need not arm itself to crush him. A vapor, a drop of water suffices to kill him. But, if the universe were to crush him, man would still be more noble than that which killed him, because he knows that he dies, and the advantage which the universe has over him; the universe knows nothing of this. All our dignity consists, then, in thought.” Pascal, *Pensées*, trans. William F. Trotter [New York: Dutton, 1958], n. 347, p. 97, cit. in [19] (p. 318).

In philosophical terms, the concept of dignity that is at stake in the principle of respect for people, as autonomous beings, which raised the bioethical declarations, was established in an exemplary way in the moral philosophy of Immanuel Kant [20]. “*Autonomy* is therefore the ground of the dignity of human nature and of every rational nature” (Kant, GMS, 2°, Ak. IV, 436), [21] (p. 42), and therefore, of the respect for oneself and others, as equally rational beings [16].

Although, as the Barcelona Declaration pointed out, the notion of dignity is broader than that of autonomy, the bond that unites both principles is so strong that they necessarily appear and disappear together due to the fact that, as Kant’s argument emphasizes, neither a dignity nor an autonomy that are ontological features of humanity are being referred to, and nor are they even the inherent condition of a self that we find has already been given. Autonomy is rather an arduous endeavor, a demanding task that has something heroic in it since it consists of striving to choose the right thing always from the appreciation and personal and direct contact with values (J. Ortega y Gasset). Hence, dignity, which is based on this constitutive autonomy (J. Rawls), which is a construction of a lifetime, is not simply a prerogative but a daily moral conquest in the face of inertia and the pressure of the impositions of what is established [16].

The following passages from *Groundwork of the Metaphysics of Morals* show in Kant the internal relationship between the universal notion of humanity which exists in the corporeal, situated, and concrete existences of each personal individuality and that of dignity, as an expression of the unique condition of end in itself, of the human being as a rational autonomous being.

“Beings the existence of which rests not on our will but on nature, if they are beings without reason, still have only a relative worth, as means, and are therefore called *things* (*Sachen*) whereas rational beings are called *persons* because their nature already marks them out as an end in itself, that is, as something that may not be used merely as a means, and hence so far limits all choice (and is an object of respect)” (Kant, GMS, 2°, Ak. IV, 428), [21] (p. 37).

“What is related to general human inclinations and needs has a *market price*; that which, even without presupposing such a need, conforms with a certain taste…has a *fancy price*; but that which constitutes the condition under which alone something can be an end in itself has not merely a relative value, that is, a price, but an inner value, that is, *dignity* […]. Morality, and humanity insofar as it is capable of morality, is that which alone has dignity” [21] (p. 42) cit. [20] (p. 334).

The reason for the dignity of each rational autonomous existence is, therefore, its possible participation in a universal legislation, but if we ask ourselves about the *what* of dignity, that is, dignified for what, then the answer is: worthy of equal respect from any other human being for the simple fact of also being a free subject of morality. Being treated as a value, as an end in itself, brings about a feeling of self-respect, which has nothing to do with self-esteem, which would be a form of self-love, but only the recognition of being co-members of a kingdom of ends in themselves, of a *noumenon* republic of free and equal beings [16]. The supreme norm of morality is therefore expressed as follows: *So act that you use humanity, whether in your own person or in the person of any other, always at the same time as an end, never merely as a means* [21] (p. 38).

However, in the notion of humanity—which comes from the Stoic tradition, especially Cicero—we also see the connection of dignity with vulnerability, with the consideration of *humanity* in every human being towards themselves and all others as an end in itself [22] (pp. 144–148). For in the notion of vulnerability it is possible to distinguish a broad sense and a strict sense that can also be found in the ethics of Kant, as has been highlighted with greater difficulty, especially in the aforementioned formulation of the moral imperative referring to humanity, since the perfect and imperfect duties, respect towards oneself as towards others, are a response both to this general vulnerable condition (broad sense) and to the specific vulnerabilities of certain people and groups (strict sense) [23]. The perfect duties to ourselves require us to always treat ourselves as ends and never just as means because our condition of rational agents as well as corporeal beings is vulnerable to different ways of abusing ourselves and lacking our own self-respect. Faced with the appeal of a duty to live, which, raised in legal terms from positions of the judiciary contrary to the normative regulation of helping to die (with dignity), which might seem inspired by Kant, it can be answered that “the right to life must be guaranteed by the political constitution, the duty to live on the other hand can only be based on a claim of the individual” [24] (p. 169).

As Oliver Sensen has pointed out, “Kant uses a fundamentally Stoic conception of dignity”. Certain passages in his work indicate that he adhered elements of what Sensen calls a *traditional* conception of dignity: (i) dignity refers to an elevation rather than a value per se; (ii) dignity allows for two stages: an initial and a fully realized conception of dignity; (iii) he connects dignity with duties, not in the first instance with rights, and (iv) he uses ‘dignity’ primarily in reference to duties towards self [25], (pp. 142, 165). So Kant shows, when facing the possibility of death by oneself, a strict and almost rigid coherence with the primacy of unconditional respect for dignity, humanity as autonomy in others and of course in oneself, such that he completely discards the possibility that Seneca left open to leave this world by his own hand in certain circumstances [21] (pp. 31–32, 38). In *Metaphysics of Morals* [II, I, 1, 1, § 5–6], Kant adds that the Stoic’s wisdom, his courage, and his greatness of mind not to fear death and to reject all the sensible motives of his conduct should be used specifically to avoid any temptation to destroy himself, an object of the utmost respect. It should be noted, as has been above, that this living by mere duty only makes sense, in any case, as a personal free choice, not as an imposition of the law.

Autonomy, insofar as it is socially constructed, through the exercise of competencies and abilities that are intersubjectively gestated, is directly exposed to certain vulnerabilities [26], and dignity is also a relational notion. This fundamentally relational conception of dignity, which shows the intertwining with vulnerability, appears in Sarah Clark Miller [27] when she considers how to understand dignity as a principle of social intervention, from the perspective of the ethics of care. She distinguishes two concepts of dignity: (i) a performative dignity, such as “a quality that can be acknowledged through how others treat us morally and also through how we treat ourselves”, which translates into an intervention model that, in contrast to the predominant Kantian conception of respect, understands care also as dignifying, as “an attitudinal recognition of another’s dignity, but one that encourages the action of the carer stepping in to support the life plans of the one for whom they are caring”; and (ii) a status dignity, “as unearned, intrinsic moral worth”, from which two possible models of social intervention from care are proposed: one in which this is conceived as “a distinctive moral power”, “the distinctive capacity that humans have to perceive, understand, adopt, and advance another person’s self-determined ends as their own”, and another in which dignity is conceived as fundamentally relational, while “care ethics is focused on the significance of relationships and the relational for our moral lives”, as highlighted by Carol Gilligan, Nel Noddings, Sarah Rudick, Eva Feder Kittay, and Virginia Held, among others [27] (pp. 112–120). This approach that it is proposed for social intervention can be perfectly applied to the field of healthcare and in particular of end-of-life-care.

A differentiation between more “individualistic”, more “relational-solidarity”, or more “secular” approaches to dignity has also been proposed to demonstrate, through research on bioethics publications in moral theology journals, that “the concept of dignity, as most of the concepts used by the science or the humanities, is not a static entity that remains identical through time and is not unanimously understood by scholars even within one domain” [28]. Michela Marzano [29] has also already made a detailed differentiation between a more individualistic and liberal version of autonomy in the utilitarian John Stuart Mill and the more intersubjective and egalitarian version of Kantian deontologism. If we now recall the explanation offered above about the close link between the principles of autonomy and dignity, which Kant proposed, we will agree that a more relational conception of dignity, that allows us to better recognize its intertwining with vulnerability, will have as a background precisely a concept of autonomy that is also more intersubjective and relational. To sum up, the conceptual constellation that includes the interweaving of autonomy and dignity, vulnerability and interdependencies is shown as the most propitious theoretical framework to address both empirical research and thinking on clinical dilemmas in end-of-life care.

## 4. Vulnerable with Dignity, towards a Paradoxical and Relational Autonomy

Vulnerable comes from the Latin term *vulnus*, wound; therefore, being vulnerable has a wide range of meanings, linked to the susceptibility of beings, bodies, and existences; to being injured, to being damaged, or one or the other. The notion of vulnerability, much more recent than that of dignity, in moral philosophy, social theory and bioethics, is equally highly polysemic and complex. Mackenzie, Rogers and Dodds (2014) have proposed a taxonomy of vulnerability, according to which, depending on the source of the vulnerability, it is possible to differentiate between: “*Inherent* vulnerability refers to sources of vulnerability that are intrinsic to the human condition”; a *situational* vulnerability, which depends on different personal, social, economic and environmental contexts, which make people or groups vulnerable. “Both inherent and situational vulnerability may be dispositional or occurrent. While the inherent–situational distinction refers to sources of vulnerability, the dispositional–occurrent distinction refers to states of potential versus actual vulnerability”. Finally, they propose a subtype of vulnerabilities: *pathogenic* vulnerabilities. “These may be generated by a variety of sources, including morally dysfunctional or abusive interpersonal and social relationships and sociopolitical oppression or injustice. Pathogenic vulnerabilities may also arise when a response intended to ameliorate vulnerability has the paradoxical effect of exacerbating existing vulnerabilities or generating new ones” [4], (pp. 7–9).

However, just as we recognized vulnerability to be inherent in dignity and autonomy, in the very core of vulnerability we find autonomy and dignity, thus, “the principle of ‘respect for human vulnerability and personal integrity’ should preferably be linked to that of ‘human dignity’, which reinforces the statement of the unconditioned value of the human beings by demanding his inviolability” [6], (pp. 161–162).

The following should also be stated: “taking ontological vulnerability seriously requires us to rethink, rather than discard, the concept of autonomy. If human persons are both ontologically vulnerable but also autonomous agents, then we need an account of autonomy that is premised on recognition of human vulnerability and an analysis of vulnerability that explains why we have obligations not only to protect vulnerable persons from harm but also to do so in ways that promote, whenever possible, their capacities for autonomy” [4] (p. 16).

From the point of view of vulnerability, it can be recognized that the dignity and autonomy towards which one aspires are inevitably paradoxical and relational in nature. Therefore, between a conception of the autonomy of rationality (Kant) and autonomy as self-determination or absolute independence of the individual in J. S. Mill’s liberalism, it is necessary to point to a third way, that of attention to capabilities. Through this, respect for the dignity of the person goes beyond acquiescence to self-determination and beyond the consideration of reason alone, and includes taking into account their history, relationships, capacities and difficulties to plan and carry out their projects, in short, their capacities and their vulnerability [30] (pp. 23–24).

For Emmanuel Levinas, the first author who philosophically addresses vulnerability [6], (p. 157), “The Self, from head to feet, to the bone marrow, is vulnerability”, constitutively the self is openness to the appeal of the Other. “Thus, in the vulnerability lies a relation to the other that is not exhausted by causality, a relation prior to all affection by the stimulus.” Vulnerability even implies an obsession with the other, with an approximation of the other, which is not reduced to the representation that the self makes of the other, nor to the awareness of their proximity [31] (pp. 62–65). When one suffers or cares for another, when one loves or hates the other, when ones places oneself in the place of another, this occurs from a responsibility that, it can be said, the self has never actively assumed but is rooted in a previous vulnerability, a “mercy”, a “commotion in the entrails”, which anticipates the responsibility that I have not assumed at any time, in any present moment” [31]. This responsibility, rooted in the vulnerability of an interdependent corporeal self, is what, together with Bernhard Waldenfels, we may call responsiveness [32].

## 5. Dignity and Vulnerability, an Ethical-Existential *Chiasma*

Article 8 of the UNESCO Declaration of 2005 states precisely that “the principle of respect for human vulnerability and personal integrity demands a new conception of the human body and disease: a body is no longer an object but a subject and hence inseparable from the person it comprises; a disease is not a purely objective phenomenon but only gains reality in a lived body and significance in the history of a life” [6] (p. 163).

The intersection that has been shown in the previous sections between dignity and vulnerability as (bio) ethical principles is based on the particular interweaving of these two dimensions of human, corporeal, social and practical existences, which would make manifest a more radical intertwining, which may be called existential. Maurice Merleau-Ponty [33] takes from anatomy the term chiasma (χίασμα, ατος, in ancient Greek, which means ‘cross arrangement’, like the letter χ) to express the peculiarity of the ‘lived body’, of a corporeal existence, which it is both sentient and sensible. The intersection of both principles (to be vulnerable and to be dignified) on the ethical-social-political and anthropological-existential levels is rooted in this chiasmatic character of the body. In addition, a certain trope or rhetorical figure is called chiasmus, which here occurs at the intersection of meanings present in the requirement to think both of a *vulnerable dignity* and a *dignified vulnerability*.

Dignity, which Pico della Mirandola associated with the capacity of a being, susceptible of becoming by choice one of the most negligible or one of the most sublime creatures, and linked by Immanuel Kant with value without equivalence, but equal to every rational-sensible being that takes for itself the norm of its action (autonomy), can also only be understood as human dignity when it is linked to the possibility of being denied, damaged or not recognized. This refers to an intrinsic vulnerability and interdependence with the human condition, in need of attention, care and response to otherness, as well as to more concrete social vulnerabilities of certain groups, populations, or, especially, difficult living conditions.

For its part, vulnerability can be thought of as an ethical principle and not as a mere condition of anthropological fragility when it is seen as a dimension of an existence capable of speaking/for itself, acting/for itself, and doing/for itself in dialogue, interaction and conflict with others, when it is transcended by the awareness of the real value of someone who is in the world, and is exposed to the world, as an agent, a co-protagonist of lives, which are intermingled in a web of stories, woven with strong ties, but always delicate and fragile.

It is due to the peculiarities of human bodily and social existence that dignity and vulnerability permeate and interpenetrate each other, in a way that is even more inseparable and indiscernible. What Paul Ricoeur has already pointed out regarding the double condition of the human being both autonomous and vulnerable could also be mentioned. On the one hand, it is the autonomy of a fragile, vulnerable being, on the other hand, fragility would not be more than a pathology, if it were not the fragility of a being called to be autonomous, because it has always been so in a certain way [34].

More than a moral principle in itself, vulnerability, as a dimension of the human condition that appeals to the attention to and care for oneself and for others, is at the very root of ethics [35,36], although it is at the intersection of dignity and vulnerability, this existential chiasma of the jointly vulnerable and autonomous human condition, where the dual active and long-suffering condition of moral subjects is best highlighted.

## 6. Conclusions

The theoretical framework of the intertwining of the bioethical principles of dignity and vulnerability in corporality that has been presented here may frame research that deals with respect for these principles in the practice of health care, from the perception of health personnel, patients and relatives. We shall analyze some studies by way of example. Approaching empirical investigations on moral questions which use the methods of the human and social sciences to elucidate ethical issues may not be justified when it comes to pure normative ethics, but this makes perfect sense when it comes to bioethics due to the eminently practical and specific nature of this area, which requires one to tread the terrain of difficult decisions in research and healthcare. From the empirical studies of health and care issues in the social and human sciences, it is possible to say what has been demonstrated in terms of the role of empirical moral psychology in bioethics, that is, that “[it] can improve bioethicists’ understanding of (1) the decision situation, (2) the origin and legitimacy of their moral concepts, (3) efficient options for implementing (legitimate) decisions, and (4) how to change and improve some parts of their moral framework” [37] (p. 36). With the help of this type of study, a radical revision of frameworks of moral concepts in bioethics can be carried out, through “actual-state, genealogical, and forward-looking analysis as well as restricted normative revision in bioethics” [37] (p. 51).

In this study on the intertwining of dignity and vulnerability as principles of bioethics, the allusion to empirical research takes on both the function of serving as an initial contrast between the relevance of the conceptual analyses that are made, as well as helping to highlight those lines of the study that seem most useful and applicable in order to understand and illuminate the experience of patients, relatives and professionals in health care, particularly in end-of-life care. The study has emphasized certain elements of analysis that help to outline a theoretical framework for future research, as well as lines of thought for a better understanding of complex health care situations in which decisions have to be made.

Human rights and public health lawyer Jonathan Mann [38] identified up to four main ways in which dignity runs the risk of feeling it has been made vulnerable in healthcare contexts, namely: a. *Not being seen*; b. *Being seen, but only as a member of a group*; c. *Violations of the bodily space*; d. *Humiliation* [10] (p. 972). Likewise, in more specific recent research on the preservation or the loss of dignity in end-of-life care in hospital emergency services [39,40], the testimonies of the participants in the study highlight the importance of contact, at least visual, between staff and patients as a sign that treatment is being received in accordance with the dignity of the person, as well as listening and empathic communication, while respecting the privacy of patients. Similarly, the professionals underline the lack of or need for time to devote not only to the most technical aspects of care but also to make it easier for patients receiving their care to feel accompanied and comfortable in their final moments of life [39] (pp. 24–26). On the other hand, the loss of dignity is associated with structural situations of the institution or the hospital department, lack of space and time, which can desensitize the healthcare worker when dealing with their patients. As some nurses participating in one of the studies stated: “Most of the time, we work without stopping, with no time to see, with no time to listen and under enough stress to make us blind to sensitivity” [40] (p. 235). “[The patient loses his/her dignity] when they stop being seen as a person in the final hours of their life, and they’re treated like a laboratory parameter that has to be improved according to repeated analytical extractions and aggressive techniques”.

Taking into account the intersection between dignity and vulnerability would help to face difficult situations that sometimes lead to paradoxical decisions with greater serenity, as highlighted by a number of studies on end-of-life care in the emergency department. Physicians show how very often the fear of families to be “alone” face to face, with the very final moments of the life of a relative, without all the hospital apparatus of personal and technical resources, coupled with a certain stubborn social mentality of the “hospital rescue”, leads to many people, even against their own will, ending their days trapped in the least conducive context for the respect for their dignity in death and feeling that they are a hindrance to those closest to them. “The family decides to take the patient to hospital, subjecting them to unnecessary tests and examinations which satisfy the popular belief that you can always do more. This means that these patients die alone, in an unfamiliar, cold, impersonal environment, where techniques take precedence over care and comfort” [40] (p. 237).

Knowing how to accompany those who are closest to us in the trance of dying, a fundamental part of caring for a worthy end of life, implies recognizing oneself sharing the feeling of the extreme vulnerability of those who see their own end near and breaking this infernal circle that some nurses identify in people they have cared for. They feel that they lose their dignity “[w]hen they show distress because of needing others to help them with their basic needs (going to the toilet, eating, etc.)” and, therefore, “[t]he patients don’t talk about how they’re feeling, I think hardly any do. They don’t want to make their loved ones even more upset” [40] (p. 237). The patient feels the vulnerability of their family members to the suffering and pain of their situation, but a wrong conception of dignity, which would exclude showing fragility, would prevent both the parent and their families from showing the necessary recognition of this common condition, and doing this would be of great help to everyone.

There are palliative care patients who are brought by the family to the emergency department because they feel overwhelmed and unable to cope with end-of-life symptoms. For this reason, some health professionals consider care that focuses on family members rather than on the dying patient. (Díaz-Cortés et al. 2018) [39] (pp. 26–27). In this context, it may be useful to remember that respect for the dignity of beings who know that they are and feel mortal is based on a notion of autonomy that recognizes the vulnerability in common and shared between those who care and those who are cared for so that the care of vulnerable beings does not result in obsessive attention or become an occasion for the imposition of paternalistic decisions.

A study with healthcare personnel [41] brought together various ways in which healthcare professionals can improve their patients’ perception of the respect for their dignity. Among others the following are mentioned: “presence” (keeping others company); “concealment” (covering up embarrassing markers of illness); “independence” (facilitating, as far as possible, patient’s self-sufficiency and moral agency); levelling (minimizing asymmetry); “creativity” (allowing patients to make or share art); “courtesy” (demonstrating common respect); and “authenticity” (honoring individuality and personhood) [10] (p. 972). 

In the study by Bovero et al. (2020) on the definition and maintenance of dignity in patients at the end of life, the responses to the question addressed to health professionals *“What comes to your mind when I say ‘Dignity’ in relation to your patients?”* were organized around the following nine items: a. “Acceptance/Listening/Attention”; b. “Respect for the person as a whole”; c. “Self-determination/Self-expression”; d. “Quality of Life (QoL)/Symptom control/Self-sufficiency”; e. “Respect for the patient’s will/wishes/needs”; f. “Privacy”; g. “Maintaining affective and social relationships”; h. “Ways of communication”; i. “Body/Care/Touch” [42]. Some of these items clearly show how expectations more related to autonomy as the nucleus of dignity (b, c, e) are mixed with those more typical of the corporeal, fragile, and vulnerable condition of patients (a, g, i).

Finally, both the studies that identify risks and losses of respect for dignity and the proposals for improving respect for dignity show that the perception of dignity is rooted in the vulnerability of bodily beings. To sum up, in care at the end of life, in coping with dying, as a limit situation of one’s own capacities to speak/for oneself, act/for oneself, and do/for oneself, in the face of the definitive threat to human frailty, which causes resentment for the whole network of interdependencies and care, the intertwining of a *dignified vulnerability* with a *vulnerable dignity* takes on a particular need to be taken into account. Talking about a dignified death is inseparable from thinking about the unavoidable experience of extreme existential and social vulnerability. A vulnerability, multiplied by the awareness of one’s own end, by knowing that the finiteness of existence is reaching its fulfillment *in me*, so that, rather than simply abandoning oneself and giving oneself entirely to the care of others, one can still rise up with pride and take upon oneself the supreme destiny with decisiveness, or anyway, expect the utmost respect for one’s last wishes or last words.

The foregoing considerations may therefore serve as an illustrative example that the clarification of the intertwining of the bioethical principles of dignity and vulnerability that is addressed here, in addition to contributing to the outline of a theoretical framework for empirical research in healthcare, can provide inspiration and ideas for decision-making and the action of patients, families and professionals in the different health care contexts, particularly in end-of-life care.

## Data Availability

Not applicable.

## References

[1] (1979). National Commission for the Protection of Human Subjects of Biomedical and Behavioral Research. Belmont Report: Ethical Principles and Guidelines for Research Involving Human Subjects.

[2] (1978). Belmont Report. Office for Human Research Protections.

[3] Rogers W., Mackenzie C., Rogers W., Dodds S. (2014). Vulnerability and Bioethics. Vulnerability: New Essays in Ethics and Feminist Philosophy.

[4] Mackenzie C., Rogers W., Dodds S., Mackenzie C., Rogers W., Dodds S. (2014). Introduction: What Is Vulnerability and Why Does It Matter for Moral Theory?. Vulnerability: New Essays in Ethics and Feminist Philosophy.

[5] Kemp P., Rendtorff J.B. (2008). Barcelona Declaration. Towards an Integrated Approach to Basic Ethical Principles. Synth. Philos..

[6] Neves M.P., ten Have H., Jean M.S. (2009). Article 8: Respect for human vulnerability and personal integrity. The UNESCO Universal Declaration on Bioethics and Human Rights. Background, Principles and Application.

[7] Rendtorff J.D., Kemp P. (2000). Basic Ethical Principles in European Bioethics and Biolaw (Vol. 1). Autonomy, Dignity, Integrity and Vulnerability.

[8] Kemp P. Final Report of the European Commission on the Project Basic Ethical Principles in Bioethics and Biolaw 1995–1998 Part B. http://cometc.unibuc.ro/reglementari/Basic-Ethical-Principles.pdf.

[9] Macklin R. (2003). Dignity is a useless concept. Br. Med. J..

[10] Andorno R. (2013). The dual role of human dignity in bioethics. Med. Health Care Philos..

[11] Andorno R. (2019). La dignidad humana como principio biojurídico y como estándar moral de la relación médico-paciente. Arbor.

[12] Nussbaum M.C. (1996). The Therapy of Desire: Theory and Practice in Hellenistic Ethics.

[13] Seneca L.A. (1925). Ad Lucilium Epistulae Morales.

[14] Nussbaum M.C., US President’s Council on Bioethics (2008). Human Dignity and Political Entitlements. Human Dignity and Bioethics: Essays Comissioned by the President’s Council of Bioethics.

[15] Zambrano M. (1994). Séneca.

[16] Cerezo Galán P. (2013). Dignidad, autonomía moral y respeto. Apuntes para una historia conceptual. Anales de la Real Academia de Ciencias Morales y Políticas.

[17] Bostrom N., US President’s Council on Bioethics (2008). Dignity and Enhancement. Human Dignity and Bioethics: Essays Commissioned by the President’s Council of Bioethics.

[18] Della Mirandola G.P. (1956). Oration on the Dignity of Man.

[19] Kass L.R., US President’s Council on Bioethics (2008). Defending Human Dignity. Human Dignity and Bioethics: Essays Comissioned by the President’s Council of Bioethics.

[20] Shell S.M., US President’s Council on Bioethics (2008). Kant’s Concept of Human Dignity as a Resource for Bioethics. Human Dignity and Bioethics: Essays Commissioned by the President’s Council of Bioethics.

[21] Kant I. (1997). Groundwork of the Metaphysics of Morals.

[22] Gerhardt V. (2019). Humanität. Über den Geist der Menschheit.

[23] Formosa P., Mackenzie C., Rogers W., Dodds S. (2014). The role of Vulnerability in Kantian Ethics. Vulnerability: New Essays in Ethics and Feminist Philosophy.

[24] Gerhardt V. (2004). Die Angeborene Würde des Menschen. Aufsätze zur Biopolitik.

[25] Sensen O. (2011). Kant on Human Dignity.

[26] Anderson J., Mackenzie C., Rogers W., Dodds S. (2014). Autonomy and Vulnerability Entwined. Vulnerability: New Essays in Ethics and Feminist Philosophy.

[27] Clark Miller S. (2017). Reconsidering Dignity Relationally. Ethics Soc. Welf..

[28] Żuradzki T., Wiśniowska K. (2020). A Data-Driven Argument in Bioethics: Why Theologically Grounded Concepts May not Provide the Necessary Intellectual Resources to Discuss Inequality and Injustice in Healthcare Contexts. Am. J. Bioeth..

[29] Marzano M. (2006). Je Consens, Donc je Suis. Etique de L’autonomie.

[30] Zielinski A. (2009). Le libre choix. De l’autonomie rêvée à l’attention aux capacités. Gérontologie et Société.

[31] Levinas E. (2003). Humanism of the Other.

[32] Waldenfels B. (2011). Phenomenology of the Alien: Basic Concepts.

[33] Merleau-Ponty M. (1968). The Visible and the Invisible. Followed by Working Notes.

[34] Ricoeur P., Ricoeur P., Pellauer D. (2007). Autonomy and vulnerability. Reflections on the Just.

[35] Kottow M.H. (2005). Vulnerability: What Kind of Principle Is It?. Med. Health Care Philos..

[36] Feito L. (2007). Vulnerabilidad. An. Sist. Sanit. Navar..

[37] Klenk M. (2020). Charting Moral Psychology’s Significance for Bioethics: Routes to Bioethical Progress, its Limits, and Lessons from Moral Philosophy. Diametros.

[38] Mann J. (1998). Dignity and health: The UDHR’s revolutionary first article. Health Hum. Rights.

[39] Díaz-Cortés M.M., Granero-Molina J., Hernández-Padilla J.M., Pérez Rodríguez R., Correa Casado M., Fernández-Sola C. (2018). Promoting dignified end-of-life care in the emergency department: A qualitative study. Int. Emerg. Nurs..

[40] Granero-Molina J., Díaz-Cortés M.M., Díaz-Cortés M.M., Hernández-Padilla J.M., García-Caro M.P., Fernández-Sola C. (2016). Loss of Dignity in End-of-Life Care in the Emergency Department: A Phenomenological Study with Health Professionals. J. Emerg. Nurs..

[41] Jacobson N. (2009). A taxonomy of dignity: A grounded theory study. BMC Int. Health Hum. Rights.

[42] Bovero A., Tosi C., Botto R., Pindincheda A., Gottardo F., Asta G., Torta R. (2020). A Qualitative Study to Explore Healthcare Providers’ Perspectives on End-of-Life Patients’ Dignity. How Can Dignity Be Defined, and Which Strategies Exist to Maintain Dignity?. J. Cancer Educ..

